# Locoregional recurrence after nephrectomy for localized renal cell carcinoma: Feasibility and outcomes of different treatment modalities

**DOI:** 10.1002/cam4.4790

**Published:** 2022-05-04

**Authors:** Yang Liu, Xinyue Zhang, Huali Ma, Li Tian, Lixin Mai, Wen Long, Zhiling Zhang, Hui Han, Fangjian Zhou, Pei Dong, Liru He

**Affiliations:** ^1^ Department of Radiation Oncology Sun Yat‐Sen University Cancer Center, State Key Laboratory of Oncology in South China, Collaborative Innovation Center for Cancer Medicine Guangzhou China; ^2^ Department of Radiology Sun Yat‐Sen University Cancer Center, State Key Laboratory of Oncology in South China, Collaborative Innovation Center for Cancer Medicine Guangzhou China; ^3^ Department of Nuclear Medicine Sun Yat‐Sen University Cancer Center, State Key Laboratory of Oncology in South China, Collaborative Innovation Center for Cancer Medicine Guangzhou China; ^4^ Department of Urology Sun Yat‐Sen University Cancer Center, State Key Laboratory of Oncology in South China, Collaborative Innovation Center for Cancer Medicine Guangzhou China

**Keywords:** recurrence, renal cell carcinoma, stereotactic body radiotherapy, surgery, systemic therapy

## Abstract

**Background:**

Locoregional recurrence after nephrectomy for localized renal cell carcinoma (RCC) is rare with diverse manifestations. The selection criteria and efficacy of different treatments are unanswered. The objective was to compare different treatment modalities and present data on stereotactic body radiotherapy (SBRT) for recurrent RCC.

**Materials and Methods:**

Patients with locoregional recurrence after nephrectomy without distant metastasis were identified from institutional big data intelligence platform between 2001 and 2020. Patients receiving local therapy (surgery or SBRT) or systemic therapy alone (targeted therapy or PD‐1 inhibitors) were divided into two groups. Progression‐free survival (PFS) and overall survival (OS) were analyzed using Kaplan–Meier method, Cox regression model. Patients were matched with propensity score matching.

**Results:**

Among 106 patients, 33 (31.1%) received systemic therapy alone and 73 (68.9%) received local therapy. Local therapy was surgery in 34 patients (32.1%) and SBRT in 39 (36.8%) patients. Patients treated with systemic therapy alone had more non‐clear cell type (*p* = 0.044), more advanced T stage (*p* = 0.006), higher number (*p* = 0.043) but smaller size of lesions (*p* = 0.042). Patients receiving local therapy had significantly longer PFS than systemic therapy (19.7 vs. 7.5 months, *p* = 0.001). After matching, the PFS in the local therapy group remained higher (23.9 vs. 7.5 months, *p* = 0.001). The 2‐year OS of the local therapy group and systemic therapy group was 91.6% and 71.8%, respectively (*p* = 0.084). Local therapy was associated with better PFS (HR 0.37; *p* = 0.0003) and OS (HR 0.23; *p* = 0.002) in multivariate analysis. Grade 2 or higher toxicities related to local therapy occurred in nine patients.

**Conclusions:**

Local therapy could delay disease progression compared with systemic therapy alone. SBRT is safe and effective for locally recurrent RCC.

## Graphical abstract

Treatment options for local recurrence of renal cell carcinoma (RCC) following nephrectomy are expanding. Our study compares different treatment modalities and elucidates that local therapy could delay disease progression compared with systemic therapy alone. SBRT is safe and effective for locally recurrent RCC.

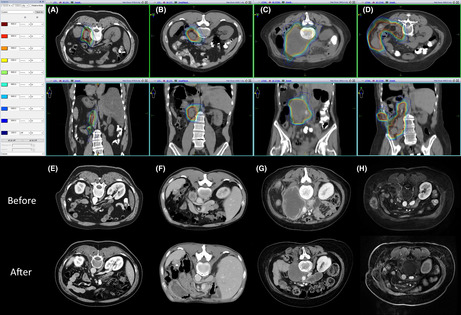

## 1 INTRODUCTION

Renal cell carcinoma (RCC) accounts globally for 3%–5% of all adult cancers, and incidence has increased steadily over the past several years.^1^ Local recurrence following nephrectomy has been reported in only 2%–6% of patients due to improving surgical techniques and adjuvant therapies. The 5‐year survival decreases to 18%–46% after recurrence, and the median cancer‐specific survival was 0.7–1 year if left untreated.^2^, ^3^ Despite the poor prognosis, generalized treatment recommendations have been left unanswered. Owing to the rarity, manifestations of this population are complex.

Salvage surgical resection is the mainstay of treatment for locoregional relapse. Surgical resection of recurrent lesions has modest effect, especially in isolated recurrence.^3^, ^4^, ^5^ In recent years, targeted agents and immunotherapy are applied to recurrent RCC,^5^, ^6^ especially for inoperable cases. Advances in radiotherapy allow highly conformal delivery of ablative doses, and an elevated dose per fraction could overcome the radioresistance of RCC.^7^ With a local control rate of 90%–98% and grade 3–5 toxicity of 0.7%–1.1%, stereotactic body radiotherapy (SBRT) has gained its place in metastatic RCC^8^ and has shown efficacy in unresectable localized RCC.^9^ In the case of local RCC recurrence, SBRT might also be effective, but the lack of data makes it staying at a theoretical level.

Treatment options for recurrent RCC are expanding, but most of the few studies have incorporated metastatic patients into analyses. Given the paucity of comparative data and absence of evidence on SBRT, the primary goal of our study was to describe and compare different treatment modalities in recurrent RCC with no distant metastasis. The secondary goal was to fill in the gap of SBRT in the management of recurrent RCC.

## 2 MATERIALS AND METHODS

### 2.1 Study population

Inclusion criteria were localized RCC with locoregional recurrence after nephrectomy. Local recurrence was defined according to the anatomic classification proposed by Lee et al.^10^ Patients with concurrent distant metastasis and recurrence confined to the remnant kidney after partial nephrectomy were excluded. Using the institutional big data intelligence platform, we identified 2414 patients with localized RCC receiving nephrectomy between 2001 and 2020. Using search terms “recurrence”, “retroperitoneal lymph node metastasis”, or “adrenal gland metastasis”, 196 records were extracted. Each record was subsequently evaluated for the inclusion and exclusion criteria.

### 2.2 Treatment

Patients were categorized into “Local therapy group” and “Systemic therapy group” according to the primary treatment they received. Patients in the “Local therapy group” received local therapy directed at recurrent lesions, including surgical excision and SBRT, either alone or with systemic therapy. Patients in the “Systemic therapy group” were treated with systemic therapy alone.

Systemic therapy was prescribed according to the guidelines at that time, so patients were treated with targeted therapy or PD‐1 inhibitors. Surgical excision was determined by surgeons based on evaluation of prior surgery, the site, and the extent of recurrent lesions. The principle of surgery was a maximal dissection of disease. For SBRT, all patients underwent 3‐mm slice thickness contrast‐enhanced computed tomography (CT) in the prone position on a belly board. Gross tumor volume (GTV) was the recurrent site. For renal fossa and/or intra‐abdominal soft‐tissue recurrence, clinical target volume (CTV) was defined as prior surgical area and/or recurrent region. For isolated recurrent abdominal lesions outside the renal fossa, CTV was equivalent to GTV. For regional lymph node recurrence, the retroperitoneal nodal region was delineated as CTV. GTV and CTV were expanded by 5 mm to generate planning target volume (PTV). Volumetric intensity‐modulated arc therapy was used for planning. Image‐guided radiotherapy was mandatory.

### 2.3 Outcomes

Patients were followed up every 3–6 months, with clinical evaluation and abdominal CT with or without chest CT scan. Progression‐free survival (PFS) was measured from the start of treatment to death or disease progression according to RECIST version 1.1. Local control (LC) was measured from initiation of local therapy to progression of treated sites. Overall survival (OS) was calculated from recurrence to last follow‐up or death. Biologically effective dose (BED) was calculated with the linear‐quadratic model, using *α*/*β* = 3.

### 2.4 Statistical analysis

Comparison of categorical and continuous variables used Chi‐squared test and Mann–Whitney tests, respectively. Univariate and multivariate analyses were performed with the Cox proportional hazards model. Only factors significant in univariate analysis were incorporated into the multivariate model. Patients with local therapy were matched 1:1 to patients receiving systemic therapy alone using propensity score matching, including all baseline characteristics as covariates. Survival was estimated by the Kaplan–Meier method. Toxicity was graded according to the Common Terminology Criteria for Adverse Events version 5.0 for SBRT, and the Clavien–Dindo classification for surgery. Statistical analyses were realized by SPSS v24 (IBM). All tests were two sided, and *p* < 0.05 was considered significant.

## 3 RESULTS

### 3.1 Patient characteristics and therapeutic strategy overview

**FIGURE 1:**
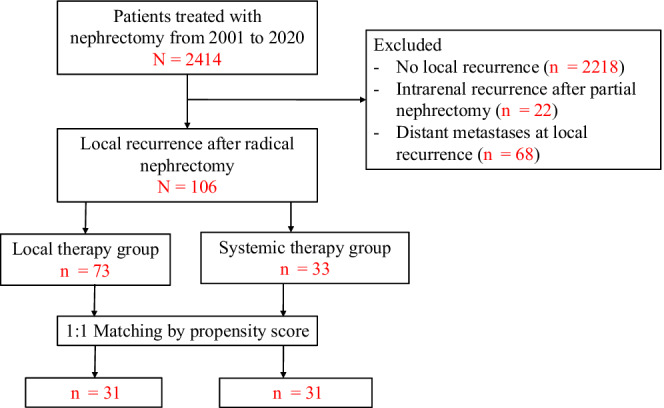
Study flow diagram

A total of 106 patients were identified, with 33 patients treated with systemic therapy alone and 73 patients receiving local therapy (Figure 1). Baseline characteristics are summarized in Table 1. Seventy‐nine patients underwent radical nephrectomy, and 27 patients underwent partial nephrectomy with only one had a history of positive surgical margin. The median time from nephrectomy to recurrence was 14.6 months. Six patients had sarcomatous features. Fifty‐two patients had non‐clear cell features with 27 (51.9%) papillary, 7 (13.5%) chromophobe, and 18 (34.6%) others. Compared with patients receiving local therapy, patients treated with systemic therapy alone tended to have non‐clear cell type (*p* = 0.044), more advanced T stage (*p* = 0.006), higher number (*p* = 0.043) but smaller size of recurrent lesions (*p* = 0.042).

**TABLE 1:** Baseline characteristics of the entire cohort and the local therapy subgroup

Features	Entire cohort (*N* = 106)			Local therapy subgroup (*N* = 73)		
	No. (%)			No. (%)		
	Systemic therapy group (*N* = 33)	Local therapy group (*N* = 73)	*p*	Surgery (*N* = 34)	SBRT (*N* = 39)	*p*
Median age, years (range)	50 (19–80)	51 (21–71)	0.344	43 (37–56)	53 (43–63)	0.039
Sex			0.058			0.929
Male	28 (84.8)	49 (67.1)		23 (67.6)	26 (66.7)	
Female	5 (15.2)	24 (32.9)		11 (32.4)	13 (33.3)	
Histology			0.044			0.224
Clear cell	12 (36.4)	42 (57.5)		17 (50)	25 (64.1)	
Non‐clear cell	21 (63.6)	31 (42.5)		17 (50)	14 (35.9)	
T stage			0.006			0.990
T1–2	10 (30.3)	43 (58.9)		20 (58.8)	23 (59.0)	
T3–4	23 (69.7)	30 (41.1)		14 (41.2)	16 (41.0)	
*N* stage			0.542			0.451
N0	23 (69.7)	55 (75.3)		27 (79.4)	28 (71.8)	
N1	10 (30.3)	18 (24.7)		7 (20.6)	11 (28.2)	
Recurrent site			0.879			0.807
Renal fossa	10 (30.3)	23 (31.5)		10 (29.4)	13 (33.3)	
RPLND ± Renal fossa	11 (33.3)	27 (37)		12 (35.3)	15 (38.5)	
Intra‐abdominal sprea	12 (36.4)	23 (31.5)		12 (35.3)	11 (28.2)	
ECOG‐PS			0.938			0.556
0–1	26 (78.8)	58 (79.5)		26 (76.5)	32 (82.1)	
2	7 (21.2)	15 (20.5)		8 (23.5)	7 (17.9)	
Number of lesions			0.043			1.000
≤5	23 (69.7)	63 (86.3)		29 (85.3)	34 (87.2)	
>5	10 (30.3)	10 (13.7)		5 (14.7)	5 (12.8)	
Size of lesion, cm (range)	2.5 (5.0–10.0)	3.5 (1.5–21.1)	0.042	3.2 (1.5–21.1)	4.0 (1.5–19.5)	0.266

Abbreviations: ECOG‐PS, Eastern Cooperative Oncology Group performance status; RPLND, retroperitoneal lymph node.

In the systemic therapy group, tyrosine kinase inhibitors (TKIs) use accounted for 29 cases (87.9%). The number of patients who received sorafenib, sunitinib, pazopanib, and axitinib was 6, 14, 6, and 3, respectively. Four patients received PD‐1 inhibitors, three of which were combined with TKIs. In the local therapy group, 34 patients (32.1%) received surgery and 39 (36.8%) patients received SBRT. Sixty‐two patients (84.9%) received systemic therapy along with local therapy. Thirty‐two patients (43.8%) started systemic therapy before local therapy and 30 (41.1%) after local therapy. TKIs remained to be the most frequently used agents, accounting for 59 cases (80.8%). The number of patients who received sorafenib, sunitinib, pazopanib, and axitinib was 8, 34, 9, and 8, respectively. Among the patients treated with local therapy alone, nine were treated exclusively with surgery, while two received SBRT alone.

### 3.2 Survival outcomes and prognostic factors

**FIGURE 2:**
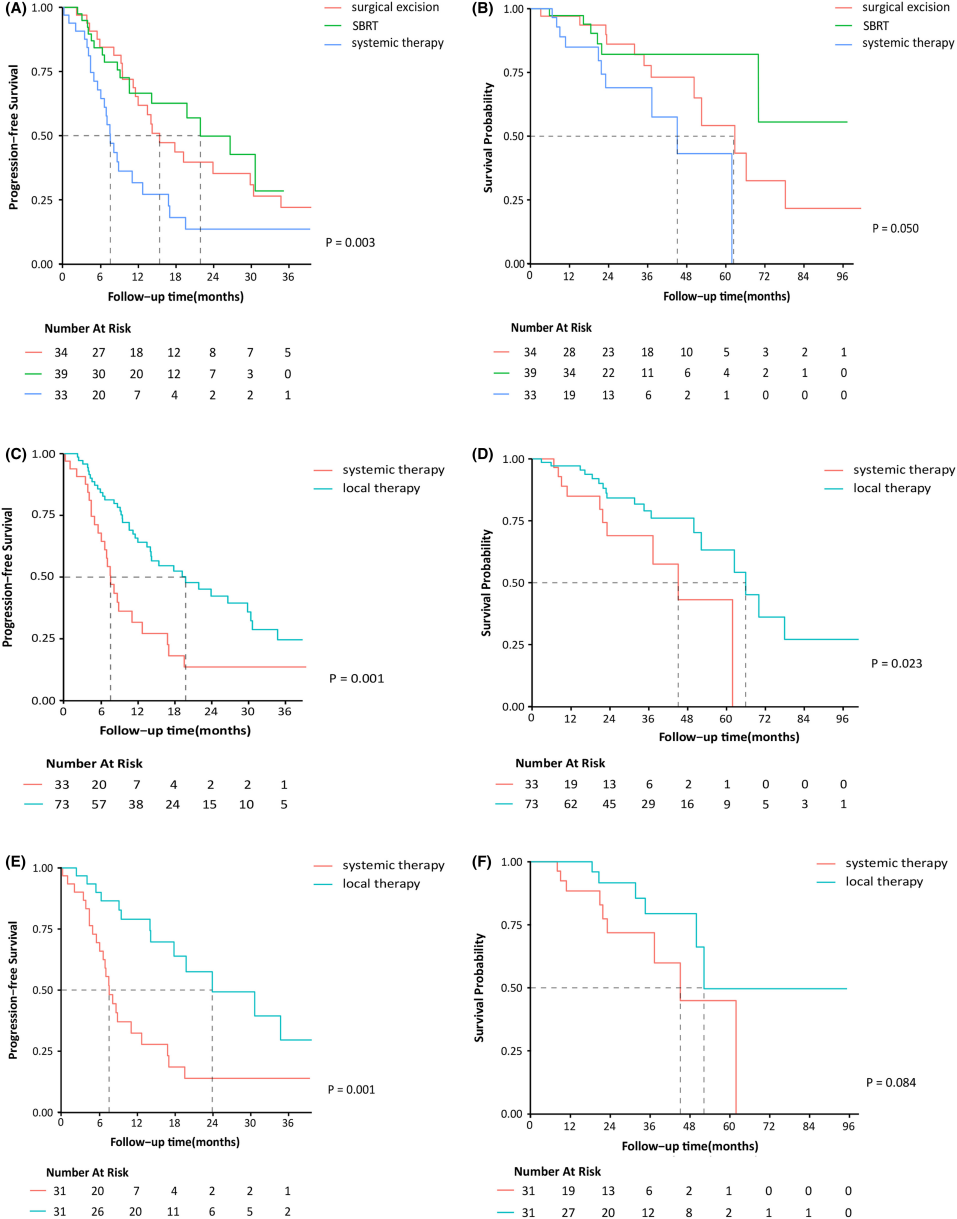
Progression‐free survival (PFS: A) and overall survival (OS: B) of patients receiving surgery, SBRT, and systemic therapy alone. PFS (C) and OS (D) of the local therapy group before matching and PFS (E) and OS (F) after matching

With a median follow‐up of 25.8 months (range, 2.8–125.1 months), 64 patients had disease progression and 28 patients died. The median PFS and OS of the whole population were 14.1 months and 62.4 months, respectively. Compared with patients in the systemic therapy group, patients in the local therapy group had longer PFS (19.7 vs. 7.5 months, *p* = 0.001). The median PFS of patients undergoing surgery, SBRT, and systemic therapy alone was 15.4, 21.9, and 7.5 months, respectively (*p* = 0.003). When comparing different local treatment modalities, SBRT and surgery yielded similar PFS (*p* = 0.523). The 2‐year OS of the local and systemic therapy groups was 84.8% vs. 69.5% (*p* = 0.023). The 2‐year OS was 86.1% after surgery, 83.3% after SBRT, and 69.5% after systemic therapy (*p* = 0.050). No significant difference was found between SBRT and surgery (*p* = 0.312). For the 32 patients who started systemic therapy before local therapy, the median PFS was 26.62 months versus 14.19 months for the 30 patients after local therapy (*p* = 0.135). The 2‐year OS was 82.9% versus 83.5% (*p* = 0.832). After matching, 31 patients in the local therapy group were matched to 31 patients in the systemic therapy group (Table S1). The PFS in the local therapy group remained higher (23.9 vs. 7.5 months, *p* = 0.001). The 2‐year OS of the local therapy group and systemic therapy group was 91.6% and 71.8%, respectively (*p* = 0.084) (Figure 2).

Results of univariate and multivariate analyses on prognostic features associated with PFS and OS are summarized in Table 2. Patients undergoing locally directed therapy demonstrated a significantly decreased risk of progression by over 60% (HR 0.37; 95% CI 0.21–0.64; *p* = 0.0003) and risk of death by around 70% (HR 0.23; 95% CI 0.09–0.58; *p* = 0.002).

**TABLE 2:** Prognostic factors of progression‐free survival and overall survival (*N* = 106)

Variables	PFS				OS			
	Univariate analysis		Multivariate analysis		Univariate analysis		Multivariate analysis	
	HR (95% CI)	*p*	HR (95% CI)	*p*	HR (95% CI)	*p*	HR (95% CI)	*p*
Histology								
Clear cell	Reference							
Non‐clear cell	1.69 (1.03–2.78)	0.040						
*N* stage								
N0					Reference			
N1					2.22 (1.01–4.91)	0.048		
Number of lesions								
≤5	Reference				Reference			
>5	1.90 (1.06–3.40)	0.032			2.53 (1.13–5.65)	0.024		
ECOG‐PS								
0–1	Reference		Reference		Reference		Reference	
>1	2.55 (1.48–4.37)	0.001	2.39 (1.29–4.41)	0.006	7.13 (3.10–16.4)	<0.001	10.2 (4.13–25.3)	<0.001
Time to recurrence								
>14.6 months	Reference		Reference		Reference			
≤14.6 months	2.13 (1.28–3.55)	0.004	2.18 (1.22–3.88)	0.008	2.56 (1.11–5.87)	0.027		
Recurrent site								
Renal fossa	Reference		Reference					
RPLND ± Renal fossa	2.18 (1.10–4.34)	0.026	2.16 (1.05–4.42)	0.035				
Intra‐abdominal spread	3.11 (1.60–6.05)	0.001	2.95 (1.49–5.81)	0.002				
Treatment								
Systemic therapy	Reference		Reference		Reference		Reference	
Local therapy	0.42 (0.25–0.71)	0.001	0.37 (0.21–0.64)	0.0003	0.40 (0.18–0.90)	0.028	0.23 (0.09–0.58)	0.002

*Note*: HR represents a 1‐unit increase.

Abbreviations: 95% CI, 95% confidential interval; ECOG‐PS, Eastern Cooperative Oncology Group performance status; HR, hazard ratio; OS, overall survival; PFS, progression‐free survival; RPLND, retroperitoneal lymph node.

In subgroup analyses, prolonged PFS was observed following local therapy in the subgroups of non‐clear cell histology, ≤5 lesions, renal fossa or retroperitoneal recurrence, and time to recurrence ≤14.6 months. As for OS, locally directed therapy was associated with a reduced risk of death in the subgroups of non‐clear cell histology and ≤5 lesions (Figure S1).

### 3.3 Characteristics of different local therapy techniques

**FIGURE 3:**
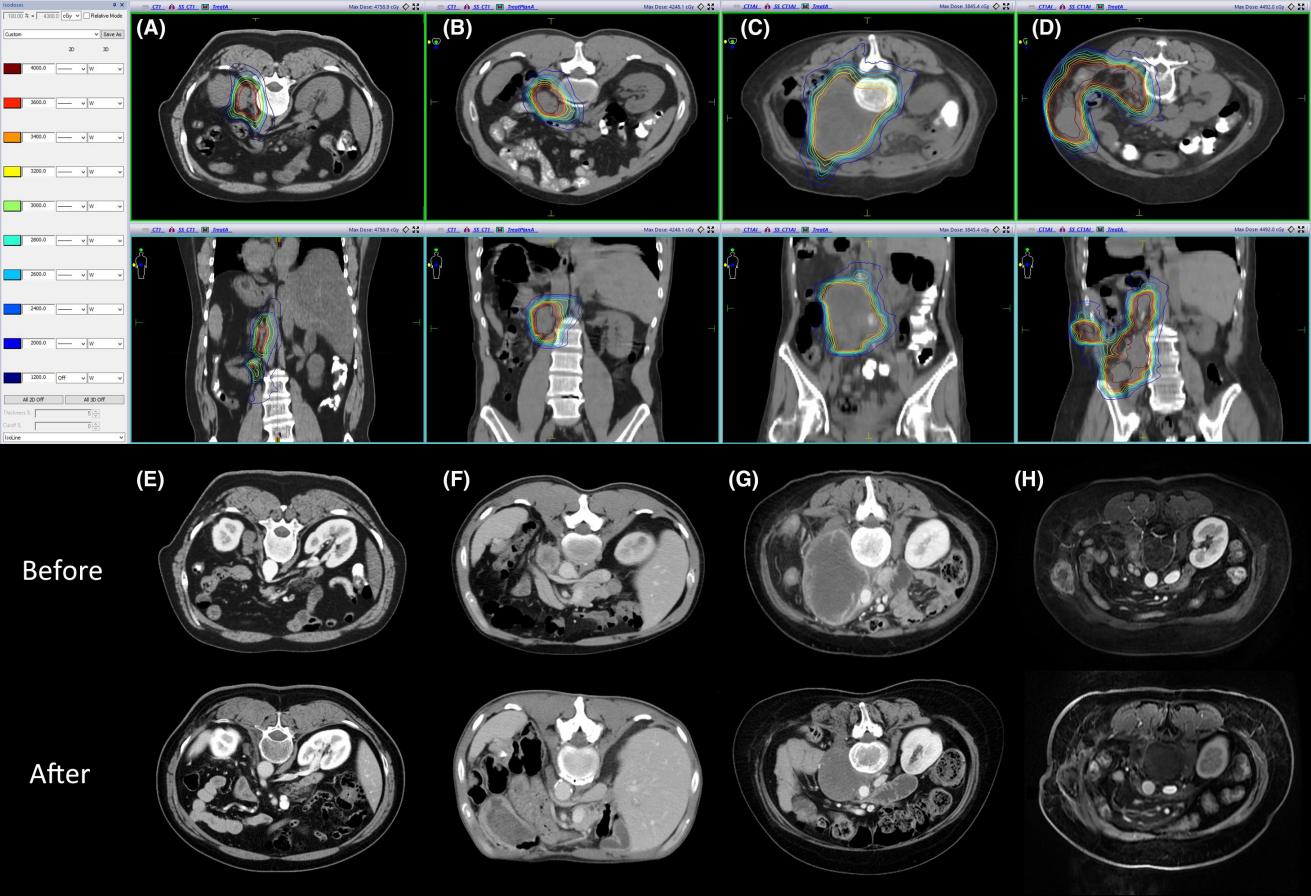
Examples of radiotherapy treatment plans and clinical response after SBRT for renal fossa recurrence (A, E), retroperitoneal lymph nodes recurrence (B, F), renal fossa and lymph nodes recurrence (C, G), and intra‐abdominal recurrence (D, H). The column on the left demonstrates the corresponding radiation dose (cGy) to each colored line

Open surgery was performed for all cases. The median surgical time was 160 min (range, 20–320 min). Median intraoperative blood loss was 100 cc (range, 5–2800 cc), and five patients required blood transfusion. In five cases, resection of an adjacent organ was performed (1 case: partial liver resection; 4 cases: colon resection). Four patients received repair of inferior vena cava (*N* = 3) and duodenum (*N* = 1) due to surgical damage. Pathological findings of recurrent sites did not differ from the histology of the initial nephrectomy. Examples of SBRT are demonstrated in Figure 3. Thirty‐three cases (87.2%) were prescribed with 30–40 Gy in 5 fractions for GTV. CTV was delineated in 33 patients (84.6%), with 20–25 Gy in 5 fractions in 29 patients (74.4%). The median BED was 117 Gy (range, 67–166 Gy) for GTV and 47 Gy (range, 40–67 Gy) for CTV. The median volume of GTV and CTV was 61 cm^3^ (range, 3–2364 cm^3^) and 386 cm^3^ (range, 96–3440 cm^3^), respectively. The dose and fraction regimen for different sites are displayed in Table S2.

Baseline characteristics were similar, except that patients treated with surgery were younger than those receiving SBRT. Twenty‐three patients progressed after surgery, 15 of which were repeated local recurrence. Seventeen patients developed progression after SBRT, all but one was infield recurrence in retroperitoneal lymph nodes. The ORR of SBRT was 48.7%. The 1‐year LC rates of surgery and SBRT were 75.1% and 96.6%, respectively.

Toxicity of grade 2 or higher related to local therapy occurred in nine patients (Table S3). In patients undergoing surgery, postoperative complications were recorded in seven patients, with one Grade I, two Grade II, three Grade III, and one Grade IV. Grade III–IV complications consisted of duodenal hemorrhage, ileal fistula, wound complications, and bleeding. In patients receiving SBRT, one patient experienced grade 3 duodenal hemorrhage.

## 4 DISCUSSION

Current data addressing the management of recurrent RCC are not conclusive and have usually lumped local recurrence with distant metastasis due to the scarcity of isolated recurrence. Thus, only systemic therapy is recommended for locoregional recurrence by current guidelines, which is inadequate given the curative potential of local therapy. Our study is a large comparative study focusing exclusively on locoregionally recurrent RCC. Our study suggested the value of local therapy in this entity and provided comprehensive data on the toxicity of this treatment modality. Besides, our study is the first research on the detailed process, effect, and safety of SBRT in recurrent RCC. This could provide some reference for further studies on radiotherapy for recurrent RCC.

Comparative studies point out that patients treated with local therapy had better survival compared with systemic therapy and expectant treatment. The median PFS and OS after locally directed therapy were around 11–23 months and 51–66 months,^2^, ^3^, ^5^, ^11^, ^12^ as well as 19.7 months for PFS and 64.9 months for OS in our study. However, the selection bias of local therapy has not been sufficiently discussed in previous comparative studies.^2^, ^3^, ^4^ Our study indicated that local therapy was preferred in patients with smaller number and larger size of lesions. This may lead to debates about whether the survival benefit was realized by local therapy. Indeed, the decision on treatment may be affected by patients' wiliness and the feasibility of local treatment. We further performed multivariate analysis and found potential survival benefits of local therapy. However, in propensity score matching, significant improvement was only observed in PFS. We did not observe improved OS like the previous study reported.^3^ Given the short follow‐up period and limited sample size, we admit that our results mainly reflect the value of local therapy in delaying disease progression, and local therapy tended to achieve longer OS without significant difference, but the results of OS are immature at present.

Local therapy is controversial due to the heterogenous survival after local therapy, emphasizing the importance of proper candidates. In our subgroup analysis, local therapy was associated with improved PFS and OS in non‐clear cell histology. Non‐clear cell RCC was more likely to develop abdominal recurrence.^13^ Since the efficacy of TKIs in non‐clear cell RCC is disappointing (ORR 5%–18%),^14^, ^15^ it is natural that the addition of local therapy had the advantage over systemic therapy alone in our non‐clear cell RCC patients. Of note, local therapy improved PFS in patients with time to recurrence <14.6 months, but no improvement of OS was found. The risk of death increased sharply as the time to recurrence decreased,^16^ indicating that patients with rapid recurrence may have an extremely poor prognosis due to prompt dissemination to distant sites. Thus, these groups of patients may be in more need of potent systemic therapy than local therapy. The current guideline is conservative in offering local therapy in quick recurrence considering poor prognosis, and our results further support this attitude.^17^, ^18^

Despite a potent approach to control locoregional disease, surgery is highly selective and individualized. Radiotherapy was less selective with respect to patients' age, functional status, comorbidity, risk profiles, and tumor location.^19^ In our study, patients receiving SBRT were older than surgical candidates, but radiotherapy was well tolerated with only two cases of grade 2 or higher toxicity. As a promising non‐invasive technique, radiotherapy has been investigated in recurrent RCC for decades, but only one case report described the use of SBRT in recurrent RCC.^20^ Maclean et al. reported a case of retroperitoneal recurrence successfully treated with CyberKnife. Other studies focused exclusively on intraoperative radiotherapy (IORT) (Table 3).^21^, ^22^, ^23^, ^24^, ^25^, ^26^ IORT alone (BED 90 Gy) led to 12%–14% in‐field failure.^21^, ^22^ With the complement of external beam radiotherapy (EBRT) (BED 157–164 Gy), the in‐field failure rate dropped to 6%–9%.^23^, ^24^, ^25^, ^26^ Meanwhile, the 1‐year LC rate was 96.6% in our patients (BED 117 Gy). These results indicate that SBRT and EBRT + IORT are both capable of providing durable local control through intensified radiation dose. However, grade 3 or higher toxicity and treatment‐related death occurred in 9%–24% and 4%–11% of patients after EBRT + IORT,^21^, ^23^, ^25^, ^26^ in contrast to 2.6% of grade 3 events following SBRT in our patients. Thus, with equivalent local control, SBRT was better tolerated.

**TABLE 3:** Summary of published literature on radiotherapy for recurrence in renal cell carcinoma

Author, year	*N*	Size of lesion, cm	Recurrent site	RT technique	RT field	IORT dose (Gy)	EBRT dose (Gy)	In‐field failure	Complications
Master, 2005	14	5 (2–17)	RF	IORT	/	15 (12–20)	/	IORT 14%	Any grade: 42% 42% ICU stay
Du, 2016	17	7	RF, Adr, RPLND	IORT	/	15	/	IORT 12%	Any grade: 37% ≥Grade 3: 24%
Hallemeier, 2012	19/22	7 (0–20)	RPLND, RF	IORT±EBRT	EBRT: TB and RPLND IORT: suspected residue	12.5 (10–20)	45 (41–55)	IORT 9% EBRT 27%	≥ Grade 3: 23% (11% Grade 5)
Calvo, 2013	10/25	4 (3–7)	RPLND, RF, IVC	IORT±EBRT	IORT: Suspected residue	15 (10–20)	40 (30–50)	IORT 9% EBRT 27% 5y‐LC 80%	≥Grade 3: 24% (4% Grade 5)
Habl, 2013	17	7 (3–14)	RF	IORT±EBRT	IORT: Suspected residue	15 (10–20)	40 (36–43)	IORT 6% EBRT 9% 2‐year LC 91%	Any grade: 24% No sever toxicity
Paly, 2014	71/98	6 (2–17)	RF	IORT±EBRT	IORT: Suspected residue	15 (10–20)	43–45	IORT 8% EBRT 9%	Any grade: 29% ≥Grade 3: 9%

Abbreviations: Adr, adrenal gland; EBRT, external beam radiotherapy; ICU, intensive care unit; IORT, intraoperative radiotherapy; LRF, locoregional failure; RF, renal fossa; RPLND, retroperitoneal lymph node; RT, radiotherapy; TB, tumor bed.

Limitations of our study included a retrospective study with a small sample size and limited follow‐up time. Secondly, patients were recruited over a span of two decades, during which time, treatment modality has been evolving rapidly. Besides, most patients in the local therapy group received systemic therapies, making it complicated to analyze the effect of each modality. Thirdly, although propensity score matching and multivariate analysis were used to minimize the imbalance of baseline characteristics, there may still be selection bias and parameters that contribute to the poor prognosis of the systemic therapy alone group. Lastly, PD‐1 inhibitors were seldom used in our study, and results may be different in the era of immunotherapy.

## 5 CONCLUSIONS

Our study indicates that in recurrent RCC patients without distant metastasis, local therapy combined with systemic therapy is tolerable and delays disease progression compared with systemic therapy alone. SBRT is a promising non‐invasive technique that may achieve similar local control to surgery with low toxicity. In light of these encouraging results, SBRT may be another local treatment option for recurrent RCC.

## AUTHOR CONTRIBUTIONS

YL, XZ, and HM participated in study design, statistical analysis, and manuscript drafting. LT, LM, and WL collected the clinical data. ZhZ, HH, and FZ contributed to the operation work during treatment. LH and PD designed the study and reviewed and revised the manuscript. All authors read and approved the final manuscript.

## CONFLICT OF INTEREST

The authors declare that they have no competing interests.

## ETHICAL APPROVAL STATEMENT

This retrospective study involving human participants was in accordance with the ethical standards of the institutional and national research committee and with the 1964 Helsinki Declaration and its later amendments or comparable ethical standards. The institutional review board of Sun Yat‐Sen University Cancer Center approved this study.

## Supporting information


Table S1‐S3
Figure S1

## Data Availability

The data that support the findings of this study are available from the corresponding author upon reasonable request.
