# Supplementary material for: Locoregional recurrence after nephrectomy for localized renal cell carcinoma: Feasibility and outcomes of different treatment modalities
Source: Cancer Med. 2022 May 4;11(23):4430–9. doi: 10.1002/cam4.4790 (PMC9741970; doi:10.1002/cam4.4790)
Supplement: Supplementary file 1 — Table S1‐S3 Figure S1 [file CAM4-11-4430-s001.docx]

Table S1. Characteristics of patients after propensity score matching (N=62)

| **Features** | **No. (%)** | | |
| --- | --- | --- | --- |
|  | **Systemic therapy**  **(N=31)** | **Local therapy**  **(N=31)** | **P** |
| Median age, years (range) | 50 (19-80) | 54 (22-71) | 0.617 |
| Sex |  |  | 0.082 |
| Male | 26 (83.9) | 20 (64.5) |  |
| Female | 5 (16.1) | 11 (35.5) |  |
| Histology |  |  | 0.442 |
| Clear cell | 12 (38.7) | 15 (48.4) |  |
| Non-clear cell | 19 (61.3) | 16 (51.6) |  |
| T stage |  |  | 0.196 |
| T1-2 | 10 (32.3) | 15 (48.4) |  |
| T3-4 | 21 (67.7) | 16 (51.6) |  |
| N stage |  |  | 0.776 |
| N0 | 23 (74.2) | 22 (71.0) |  |
| N1 | 8 (25.8) | 9 (29.0) |  |
| Recurrent site |  |  | 0.459 |
| Renal fossa | 10 (32.3) | 10 (32.3) |  |
| RPLND ± Renal fossa | 10 (32.3) | 14 (45.2) |  |
| Intra-abdominal spread | 11 (35.5) | 7 (22.6) |  |
| ECOG-PS |  |  |  |
| 0-1 | 24 (77.4) | 27 (87.1) |  |
| 2 | 7 (22.6) | 4 (12.9) |  |
| No. of lesions |  |  | 0.562 |
| 1-5 | 22 (71.0) | 24 (77.4) |  |
| >5 | 9 (29.0) | 7 (22.6) |  |
| Size of lesion, cm (range) | 2.6 (0.9-10.0) | 3.5 (1.5-19.5) | 0.348 |

ECOG-PS, Eastern Cooperative Oncology Group performance status; RPLND, retroperitoneal lymph node

Table S2. Dose and fraction regimen of SBRT for different recurrent site (N=39)

| **Location** | **No. (%)** | **Fractions** | **GTV median dose, Gy (range)** |
| --- | --- | --- | --- |
| Renal fossa | 1 (2.6) | 4 | 32 |
|  | 12 (30.8) | 5 | 35 (28-43) |
| RPLND ± Renal fossa | 15 (38.5) | 5 | 35 (25-40) |
| Intra-abdominal spread | 11 (28.2) | 5 | 40 (30-43) |

Table S3. Toxicity of grade 2 or higher following local therapy

| **Toxicity description** | **Grade** | **Surgery** | **SBRT** |
| --- | --- | --- | --- |
| Anorexia | 2 |  | 1 |
| Duodenal hemorrhage | 3 | 1 | 1 |
| Duodenal obstruction | 2 | 1 |  |
| Bowel obstruction | 2 | 1 |  |
| Lymphorrhagia | 2 | 1 |  |
| Ileal fistula | 3-4 | 2 |  |
| Wound complications | 3 | 3 |  |

Figure S1. Subgroup analyses of progression-free survival and overall survival.
